# Typical MRI Imaging with Clinically Mild Encephalitis/Encephalopathy of a Reversible Splenial Lesion (MERS) Caused by Influenza A Virus

**Published:** 2020-01

**Authors:** George IMATAKA, Shigemi YOSHIHARA

**Affiliations:** Department of Pediatrics, Dokkyo Medical University, Tochigi, Japan

## Dear Editor-in-Chief

We report a case of acute encephalitis associated with transient MR signal intensities in the splenium of the corpus callosum (MERS; clinically mild encephalitis/encephalopathy with a reversible splenial lesion) caused by influenza A virus.

The case was a 6-yr-old girl was referred to Dokkyo Medical University Hospital, Shimotsuga, Japan for persistent coma after cluster febrile seizure in winter season. Using rapid antigen test, she was diagnosed with influenza type A infection 6 days earlier.

After we obtained informed consent from parents of the patient, she admitted our hospital and performed both examination sand treatments. Cerebrospinal fluid test showed increased cell count (188/mm^3^), elevated protein (68 mg/dl) as well as markedly high level of interleukine-6 (14,200 pg/ml). IgG index was 0.86 at this time. Diffusion weighted magnetic resonance image (MRI) showed increased intensity in the splenium of the corpus callosum. Other MR findings also including ADC-map, FLAIR, T1 and T2-weighted images revealed abnormal intensities in the splenium.

Disturbance of consciousness state never recovered, and she was treated with methylprednisolone steroid pulse therapy (30 mg/kg×3days) as well as high dose immunoglobulin therapy (400 mg/kg×5days). At the 3^rd^ day of the dual treatment, her conscious level gradually recovered, and completely recovered on the 13th day from the onset. There were no neurological sequelae following her discharge from the hospital after one year from the onset.

**Fig. 1:**
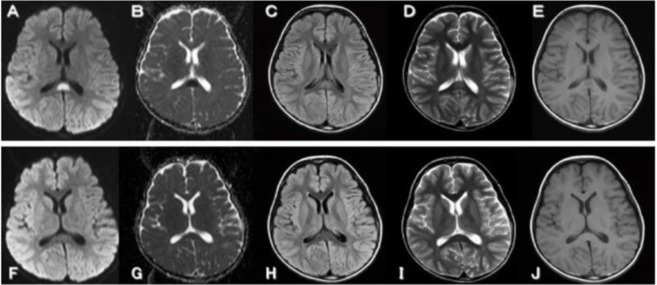
Brain magnetic resonance (MR) imaging on day 7 from the onset, showed abnormal signal intensities in the splenium of the corpus callosum: markedly high signal intensities on diffusion-weighted imaging (DWI) : (A), and markedly low signal intensities on an apparent diffusion coefficient (ADC) map : (B), slight hyperintensity developed in the same area on fluid-attenuated inversion recovery (FLAIR) : (C: TR=9000 / TE=105) and on fast spin-echo T2-weighted image : (D: TR=3800 / TE=99). Otherwise on spin-echo T1- weighted images showed mild decreased intensity signal in the splenium: (E: TR=593 / TE=15). MR imaging findings on day 13, demonstrating completely resolved of these abnormal intensities on DWI: (F), ADC map: (G), FLAIR : (H), T2-weighted image : (I) and T1-weighted image : (J)

MERS is an acute encephalopathy with decent prognosis reported from Japan. There are often subsequent occurrences of influenza type A infection. MRI-diffusion weighted imaging is a powerful tool for diagnosis for this condition. Occasionally aihigh signal is observed on T2-weighted image and contrast image showed no effect. Some previous reports also reported MERS mainly recognized diffusion-weighted imaging (1–3). Our case demonstrated abnormal signal intensities in the splenium of the corpus callosum: markedly high signal intensities on diffusion-weighted imaging (DWI) (Fig. 1). Moreover, these abnormal intensities were completely disappeared. The reasons for these abnormal splenial lesions were considering with cytotoxic edema or edema in the myelin sheath (4). At the beginning, her clinical course was not mild and revealed continue comatose. During the steroid pulse and immunoglobulin treatment, her consciousness level was gradually recovered. Her clinical course could have been a mild type of MERS and the case was very interesting with regard to consideration of the mechanism of the unique encephalitis in transient MR intensities in MERS.
